# Supplementary material for: A Bispecific Antibody Blocking Both TSLP and IL-4Rα for the Treatment of Allergic Inflammatory Diseases
Source: Cells. 2025 Nov 7;14(22):1747. doi: 10.3390/cells14221747 (PMC12651572; doi:10.3390/cells14221747)
Supplement: Supplementary file 1 [file cells-14-01747-s001.zip › cells-3928818-supplementary.pdf]

SUPPLEMENTAL FIGURE S1

(A)

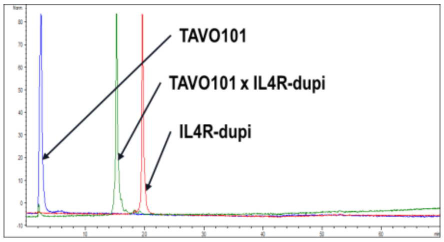

(B)

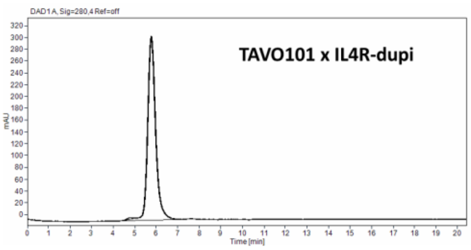

(C)

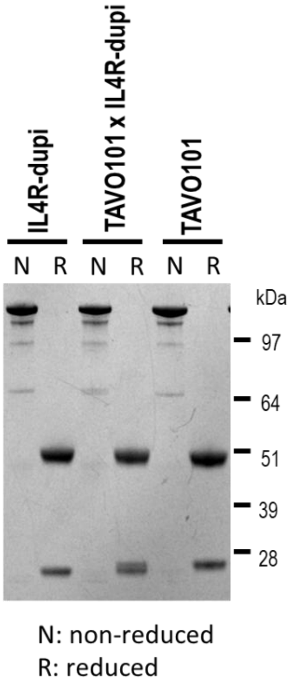

Figure S1. Characterization of TAVO101 × IL4R-dupi BsAb. (**A**) CEX analysis of TAVO101 × IL4R-dupi and the corresponding parental antibodies TAVO101 and IL4R-dupi. The Y axes units are normalized peak absorbance at 280 nm. The x axes units are retention time in minutes. (**B**). SEC analysis of BsAb TAVO101 × IL4R-dupi. The Y axes units are in absorbance values at 280 nm. The x axes units are retention time in minutes. (**C**). Gel images of protein bands of TAVO101 × IL4R-dupi and the corresponding parental antibodies TAVO101 and IL4R-dupi, subjected to SDS-PAGE analysis under reduced and non-reduced conditions.

SUPPLEMENTAL FIGURE S2

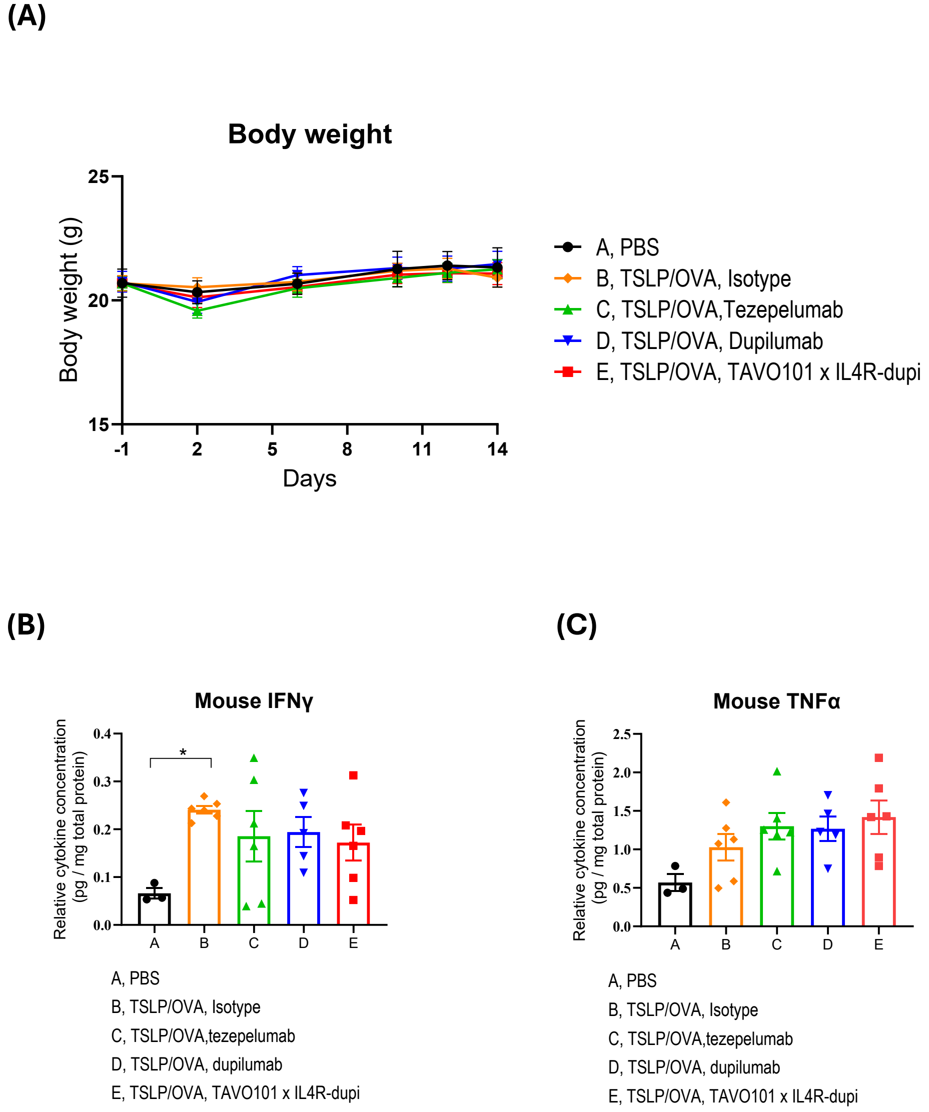

Figure S2. Efficacy of TAVO101 × IL4R-dupi in a TSLP/OVA-induced asthma model. (**A**) Mouse body weight changes for each group over the study period. (**B**). Bar graph showing the concentration of mouse lung tissue IFNγ in each group. (**C**). Bar graph showing the concentration of mouse lung tissue TNFα in each group. Data was represented by mean ± SEM.
